# Qualitative views of Nigerian school principals and teachers on the barriers and opportunities for promoting students’ physical activity behaviours within the school settings

**DOI:** 10.1186/s12889-021-12327-x

**Published:** 2021-12-19

**Authors:** Mojisola Morenike Oluwasanu, Oladimeji Oladepo, Segun Emmanuel Ibitoye

**Affiliations:** Department of Health Promotion and Education, Faculty of Public Health, College of Medicine, University of Ibadan, Ibadan, Nigeria

**Keywords:** Physical inactivity, In-school adolescents, Behaviour, Sports, Students

## Abstract

**Background:**

Insufficient physical activity (PA) is a growing public health challenge among Nigerian adolescents. Significant information gap exists on the school-related factors which influence the participation of adolescents in school-based physical activity programmes in Nigeria. This study was conducted to document the qualitative views of school principals and teachers on the barriers and opportunities for promoting the physical activity behaviours of adolescents within the school settings in light of the socio-ecological model.

**Methods:**

This was a qualitative study conducted in 12 public and private schools in two local government areas of Oyo state, Nigeria*.* Two key sources and data collection methods (i.e key informant interviews and focus group discussions) were used. Six key informant interviews were held with school principals and six focus group discussions with classroom teachers using pre-tested guides. Data was analysed using thematic analysis.

**Results:**

Fourteen sub-themes were identified as barriers to PA and linked to different levels of the socio-ecological model. Three themes were categorised as parental factors, three themes as socio-cultural and religious factors while the school-related factors had eight sub-themes. Specifically, the school-related barriers were the declining number of trained physical health education teachers, limited opportunities for continuing education and low prioritisation of physical health education. Other school-related factors such as increasing demand for classroom academic time, negative attitudinal dispositions of other teachers and inadequate funding for schools which hampered the provision of facilities and equipment were identified as factors that limit the effective implementation of policies and programmes for physical activity in schools. Opportunities to promote PA within the school settings during assemblies, breaktime, after-school and inter-house sports competition exist. However, these opportunities are hampered by competing academic time, security threats, fear of causalities to students due to poor supervision after school, poor funding and brawling associated with competitive school-based sporting events.

**Conclusions:**

Factors that contribute to insufficient physical activity among in-school adolescents in the school settings are multi-factorial. Implementation of holistic, multi-component interventions which address the social-cultural and school-level factors and enhance students’ opportunities for physical activity in schools are recommended.

**Supplementary Information:**

The online version contains supplementary material available at 10.1186/s12889-021-12327-x.

## Background

Physical inactivity in childhood and adolescence has been linked to the increased risk of cardiovascular diseases, cancers, diabetes mellitus, obesity, anxiety and poor emotional wellbeing [1–4]. In 2018, the World Health Organisation modified the Global Action Plan on Physical Activity (GAPPA) and set a goal of 15% decrease in prevalence of physical inactivity among adolescent [5]. However, 81.0% of students globally (77.6% in boys and 84.7% in girls) remain physically inactive further rendering a setback to the targeted 15% global adolescent physical inactivity reduction by 2030 [6]. Studies in different regions of Nigeria have documented a high level of physical inactivity among Nigerian adolescents [7–11]. A study conducted in Ibadan, South-western Nigeria revealed that over a third of the in-school adolescents (38%) were not involved in sufficient physical activity (PA); 58.8 and 3.2% engaged in low and high intensity physical activity behaviours respectively [7]. In another study, in Ibadan, more than half of school-going adolescents reported low levels of PA [8]. A study in a rural setting in Oyo state, South-western Nigeria showed that approximately a third of the students had insufficient PA [9]. Oyeyemi et al. found that only 37% of in-school adolescents in Maiduguri in the North eastern geopolitical region of Nigeria attained 60 min of moderate and vigorous physical activity (MVPA) daily [10]. Physical inactivity has also been reported among adolescents in Ogun state in the South-western region of Nigeria [11, 12].

Systematic reviews and research studies in other parts of the world have revealed that multiple factors such as individual, parental and school-level factors determine the physical activity behaviours of adolescents [13–17]. These findings align with the principle of the socio-ecological model which acknowledges the intricate relationship which exists between an individual and their social, physical and policy environments. The social-ecological model is a broad framework which emphasises the unique complexities and intricate relationship existing between an individual and the social, physical and policy environments [17].

The model posits that individuals are responsible for implementing lifestyle behavioural changes critical for reducing health risk and improving health, but their ability to implement the proposed behaviour is determined largely by the external environment e.g. interpersonal factors, community norms and values, institutional factors, regulations, and policies. This model has been adapted to critically analyse the factors influencing the physical activity behaviours of adolescents [17]. In this study, perceived parental factors, religious and socio-cultural factors and the influence of the schools’ external environment (PA Policies, rules, regulations, guidelines, infrastructure availability) on students’ physical activity patterns were the major focus.

Studies in Nigeria have shown that individual factors such as age and sex were determinants of PA behaviour. The study by Adeniyi et al. in Ibadan revealed that the mean score for physical activity was higher among younger in-school adolescents (3.1 ± 0.8) than older adolescents (2.2 ± 0.5) [9]. Similarly, Oyeyemi, Ishaku and Oyekola et al. in their study in northern Nigeria revealed that moderate-intensity and total physical activity was lower in older in-school adolescents in Maiduguri than in the younger group [10]. Sex was also a critical factor which influenced PA behaviour. Girls on the average spent approximately 23 min/day lower time in MVPA compared to boys (60.6 min/day versus 83.4 min/day) [10].

Nigeria is a multi-ethnic, multi-religious country and this shapes the culture of the country; the largest ethnic groups are the Hausa and Fulani in the north, Yoruba in the southwest and Igbo in the southeast. This cultural expression influences the gender and traditional cultural roles and may be a key influence for the PA behaviours of adolescents. For instance, Oyeyemi et al. found that Nigerian adolescent girls spend more time than boys in domestic and light-intensity physical activities while boys spend more time in leisure-time and vigorous-intensity activities than girls [10]. Another study in Ogun State, south-western Nigeria found that adolescents believed it is not safe for girls to participate in physical exercise and this can easily make a girl masculine and her breast to sag [11]. There is a dearth of information on the influence of socio-economic status on PA, however available data shows that adolescents from households with higher socio-economic status reported more MVPA and total PA compared to those from households with lower socio-economic status [10] though another study reported no association [12].

Some studies in Nigeria have also revealed the influence of school-level factors on PA. The 2018 Report Card on Physical Activity for Children and Youth revealed that 40% of children in public schools and 80% in private schools engage in organised sports [18]. Most schools in Nigeria offer the physical health education subject to pupils in the junior secondary school (10 to 13 years age group) as part of the curriculum two to three times per week. The maximum allotted time is 40 min per class which is devoted largely to class room academic activities [19]. It is offered as an optional subject for pupils in the senior secondary level (14 to 17 years age group) though it is compulsory for the junior secondary level [20]. However, majority of pupils at the senior secondary schools do not enrol for the course since it is optional for their level [20]. The number of students in PA lessons per week varies from 20 or less to over 50 or more depending on the population of students in each school. Generally, public schools have more pupils per class compared to private schools [Personal Communication with Local Inspector of Schools, Ministry of Education, Oyo State, Nigeria].

In addition, though most secondary schools in Nigeria offer physical health education as part of the educational curriculum, they have limited equipment, personnel or space to implement the physical activity programmes [18]. Nigeria has some policies which are expected to influence PA in schools. These include the *Sports Policy (2009), the School Health Policy (2006) and the* National Multi-Sectoral Action Plan for the Prevention and Control of Non-communicable Diseases (2019–2025) [21–23]. These policies have outlined interventions to increase the physical activity levels of the populace, adolescents inclusive. Specific activities in these policies are “*to support development of physical health education and sporting activities in schools* [21]*; ensure sports is offered as an obligatory subject at the primary and secondary school level; ensure all Nigerian primary and secondary schools have grounds for play and sporting activities which will be a condition precedent for approval for schools’ establishment* [22]*, implement school-based programmes in line with WHO health-promoting school initiative* [23]*”* however, comprehensive intervention remains low [24].

Schools are ideal settings for influencing the physical activity behaviours of young people [25, 26]. These provide important social and physical environments where students are inculcated with knowledge and skills which enhance their abilities to observe, model and practice healthy behaviours. In Nigeria, secondary education is mostly provided by the state and federal government at free or subsidised rates however, there is a growing increase in private-owned secondary schools though these cater largely to the educational needs of adolescents from the upper- and middle-class households while adolescents from largely low-and low-middle income households attend public schools. In-school adolescents spend the greater part of their waking hours in schools with various opportunities for PA such as break times, after the school day and physical education lessons [4, 27–30]. Thus, they have great potentials to influence the physical activity behaviours of students [19]. Furthermore, school-based physical activity provides an ideal ecological approach to the design and implementation of holistic behaviour change interventions [17, 31].

Globally, there is a call to address the underlying causes of physical inactivity by implementing population- level interventions in health promoting settings such as workplaces and schools [4]. Considering that in-school adolescents spend up to 8 h at school and attend several after-school programmes, it is apt to assess the potential barriers or opportunities in schools which influence the physical activity behaviours of in-school adolescents.

Though a number of research studies on the physical activity behaviours of young people have been conducted in Nigeria, most of these have focused largely on individual behaviours [7–12] without critical qualitative exploration of the influence of the school settings on their physical activity patterns. Focusing on individualised behaviour change interventions, instead of a systems approach [4] premised on the socio-ecological model adapted for physical activity by Sallis et al., [17] reduces the opportunity of maximising the effectiveness of interventions. Thus, this qualitative study was conducted to identify barriers and opportunities for promoting the physical activity behaviours of in-school adolescents in Oyo state, Nigeria in light of the socio-ecological model. This may contribute to the future design and implementation of interventions and policies which potentially may improve the physical activity behaviors of adolescents.

## Methods

The data presented in this article is part of a larger study titled “*Effects of a multi-level intervention on the pattern of physical activity among in-school adolescents in Oyo state Nigeria: a cluster randomised trial*”. The study protocol and data collection procedures have been reported in a previously published article [32]. This article presents the qualitative findings from 12 schools randomly selected out of the 22 schools recruited for the main study after stratifying by school ownership (public or private). The cluster randomised trial was conducted in two urban local government areas hence; the schools selected for the qualitative interviews were located in urban settings. For the selection of schools for the cluster randomised trial which is the main study, 22 schools were selected using a multi-stage sampling method. There were more public schools because they had a larger population of secondary school students compared to the private schools. From the pool of 22 schools selected for the main study, (approximately 50%) 12 were selected for the qualitative data collection due to funding constraints.

### Study areas and population

The study areas were two Local Government Areas (LGAs) located in two urban cities in Oyo State, south western Nigeria. Oyo state is inhabited predominantly by the Yorubas though there are several other ethnic groups such as the Igbos, Hausa and other minorities. Ibadan North-west LGA is located in Ibadan while Ogbomosho North LGA is located in Ogbomosho. Ibadan North-west LGA has 13 public and 18 private secondary schools while Ogbomosho North LGA has 15 public and 26 private secondary schools. The selected schools for this study have a student population ranging from 152 to 1417 with more students in the public secondary schools.

The study population for the qualitative phase of the study were School Principals and Teachers in 12 schools (comprising eight public and four private schools in both LGAs) [Additional file 1]*.* Two key sources of data were used (i.e. interview, focus group discussions). We had six key informant interviews with School Principals (three each from each LGA) and six Focus Group Discussions with teachers who teach different subjects including physical health education (three each from each LGA). The complementary use of the focus group discussions and key informant interviews was done to enrich our understanding of the qualitative views of school stakeholders about the physical activity behaviours of adolescents and influencing factors. Denzin [33] described this approach as methodological triangulation and the specific method used was the blended design which implies the combined use of qualitative designs which in this study were the focus group discussions and key informant interviews [33, 34]. This approach has been used in public health studies [35, 36].

### Instruments

The study tools [see Additional files 2 and 3] were developed using constructs from the socio-ecological model adapted for physical activity by Sallis et al. [17]. The key informant interview guide for School Principals assessed their perspectives about the physical activity behaviours of secondary school students in their schools, frequency of engagement in structured and unstructured physical activity and factors (social-cultural, built environment and policy) which influence the physical activity behaviors of the students, the extent to which school guidelines and policies provide opportunities for PA, barriers to the implementation of school physical activity policies and programmes *(PHE curriculum, annual inter-house competition where students in the same school are organised into groups and compete against each other to win medals and opportunities for PA* e.g. *school assembly, break-time/recess etc*) in schools, level of funding and recommended activities to ensure in-school adolescents attain the daily target of 60 min of moderate and vigorous daily activity.

The focus group discussion guide was used to assess teachers’ opinion about the frequency and opportunities for engagement in structured physical activity (i.e. *sports, class based activities, regular school physical activity programmes*) as well as unstructured activities (i.e. *break time (leisure), after school programmes* etc.), social support for physical activity, barriers for the implementation of physical activity policies and programmes *(PHE curriculum, and school time table/term/annual plans which indicate timeline for inter-house competition and other PA activities* e.g. *school assembly, break-time/recess etc*) in schools and feasible interventions. The participants were teachers who teach different subjects including physical health education. In Nigeria, all teachers irrespective of the subject which they teach are assigned classes to manage. Since the researchers were interested in the participants’ experience of the students’ school-based PA participation, it was important to include several teachers irrespective of the subject they teach provided they have contact with students. In addition, some schools did not have PHE teachers and where they had, they were limited and may not have a comprehensive perspective of the students’ PA participation within the school setting.

### Data collection

Data collection spanned 4 weeks, two public health professionals with previous training and experience in qualitative research methods conducted the interviews and focus group discussions and a note taker documented the key points from the discussion verbatim.

Six key informant interviews (KII) were held with Principals/Vice principals in selected public and private schools (3 schools each in Ibadan and Ogbomosho). The key informant interviews were held within the school premises at comfortable venues selected by participants, free of distraction and noise. The duration for the KII session ranged between 25 to 40 min.

Six focus group discussions (FGD) were held with teachers in selected public and private project schools (3 schools each in the Ibadan and Ogbomosho). Participants’ names were not obtained but number was assigned to them for easy identification. The focus group discussions were held within the school premises at comfortable venues free of distraction and noise. Each FGD session had a group of discussants ranging from six to ten and the duration of the sessions ranged between 40 to 55 min.

### Data analysis

The interviewers who conducted the interviews transcribed the audio recordings verbatim to word files. Data quality checks were ensured throughout the data collection and transcription of the interviews. The research team (MMO and SEI) listened to the interviews and compared to the transcription to identify errors/discrepancies found in the data. The transcribed data was cleaned and saved in word format. Identification codes were assigned to all individual records including audiotapes, transcripts and demographic information. Tentative a prior themes [Additional file 4] were identified based on the research objectives and how often some features reoccurred in the data but these were modified to capture memorable and useful outliers comments which may aid the understanding of the data. These themes guided coding and data analysis. The interview transcripts were loaded into NVIVO 10 for coding and data analysis was done using thematic analysis guided by the study objectives. A public health professional with over 15 years of qualitative experience led the analysis and two trained independent coders with background in public health coded the data. The lead public health professional independently cross checked the data coded to ensure it aligned with the study objectives and themes. There were debriefing meetings by the research team members and data coders to read and re-read the transcripts, discuss the data findings and identify /modify themes to guide the coding of the data and the analysis process. After data analysis, the preliminary findings were presented at a meeting to representatives of all schools who participated in the study and the findings were adjudged by participants to reflect their views and perspective on physical activity in the school setting.

### Ethical issues

The study followed ethical guidelines for studies involving human subject research. This study received ethical approval from Oyo State Research Ethical Review Committee (AD13/479/890) and permission from the Oyo State Ministry of Education. The Local Inspectors of Education of the local government areas and School Principals also provided permission for the conduct of the study. Teachers and Principals interviewed provided written consent for participation and publication of the findings. The participants were provided detailed information on the objectives of the study and assured of their confidentiality and other ethical issues. Request for permission to use digital voice recorded was made and written consent was obtained from all the discussants.

## Results

### Demographic characteristics

Six School Principals/Vice Principals participated in the key informant interviews while 53 teachers participated in the focus group discussion sessions. Most of the key informant interviewees were males (66.7%) while the focus group discussants were largely females (56.6%). The ages of the key informant interviewees ranged from 45 to 57 years (mean age = 49.3 ± 4.1) while for the focus group discussants, it ranged from 23 to 45 years (mean age = 39 ± 8.7).

### Perceived parental factors influencing the physical activity behaviors of adolescents

This theme refers to the perceived parental factors in the family microsystem which influence the physical activity behaviours of adolescents. It comprises three sub themes: parental education and participation in PA, cultural beliefs on the effects of sports on females’ future fertility potential and preference for motorised transportation rather than active commuting for their children/wards. The prominent sub-themes were parental education and participation in PA and cultural beliefs on the effects of sports on females’ future fertility potential.

### Parental education and participation in PA

This was a prominent sub-theme and participants expressed that parental level of education had implication on their appreciation of the importance of PA and its effect on the health of their children. They were of the view that parents had limited knowledge on the importance and benefits of physical exercise which invariably influenced their ability to encourage adolescents to engage in PA as expressed in the quote by a discussant in a private school *“The level of education of the parents will determine the extent to which they will appreciate physical exercise. Our parents* “(...)“ *do they know the importance of physical exercise? do they encourage the adolescents to participate in PA? do they appreciate it? the answer is no”* (FGD discussant from a Private Secondary School). Similar views were expressed by a key informant from a public school who stated that parental illiteracy contributed to poor awareness of the benefits of PA among adolescents and this had implication on parents’ involvement in PA and their ability to model this healthy behaviour. This finding is illustrated in this quote ***“****Majority of the adolescents are not aware of the benefits of physical fitness and their parents are illiterates. They [their parents] don’t participate in any exercise*” (Key Informant from a Public Secondary School).

### Parental cultural beliefs on females’ participation in sports and its effect on fertility

This was also a major sub-theme, participants expressed that parents’ cultural beliefs on the effects of female’s participation in sports on future fertility was a major influence on their physical activity behaviours. This finding is a reflection of the general, prevailing societal belief and was expressed by participants as a barrier to PA as summarised in this quote *“Some parent prevent their female children or wards from participating in sporting activities because they believe that they would not be able to give birth to a child later on, so they prevent them from the activities”* (FGD discussant from a Public Secondary School).

### Parental preference for motorised transportation

Active commuting is a feasible approach to increase PA participation among adolescents however; most parents in both the public and private schools prefer motorised transportation which is likely linked to the poor road network and increasing spate of roadside accidents and injuries. The participants expressed that parental preference for motorised transportation rather than active commuting was a barrier to PA and opined those parents were indulging their children making them dependent on motorised transportation as expressed by a key informant **“***many parents are indulging their wards/children. Instead of encouraging them to walk, they prefer to give them money to take “okada” [motor bicycles] or to take taxis [cabs]”* (Key informant from a Public Secondary School).

### Socio-cultural and religious factors influencing physical activity behaviors of adolescents

This theme focuses on the macro-level socio-cultural and religious factors which influence the physical activity behaviors of adolescents. It comprises two prominent sub-themes which are, the cultural limitations on females’ participation in sports and its implication on their health and marital prospects and cultural limitations on the level of interaction between the opposite sex. The less prominent theme which emanated from the data was the religious injunctions on females’ dressing.

### Cultural limitations on females’ participation in sports and its implication on their health and marital prospects

Majority of participants expressed prevailing socio-cultural perspectives and misconceptions which limit the participation of females in PA. Specifically, they stated that the society discourages PA in females because their body is delicate and can easily be harmed. In addition, they stated that some people believe that involvement in vigorous physical exercises can break the hymen of females and also result in the development of masculine features. These observations according to the views expressed by the participants can also cause infertility in females or limit their opportunities to get married in the future as illustrated by the quote from a FGD discussant from a private school *“some people believe that if a virgin exercises too often, she will become disvirgined. If they run too much* “(...)” *it can make them look more like a man and they will develop muscles. So, they discourage their female children that it makes them look like a man and they ask, who will marry you?”*

### Cultural limitations on opposite sex interaction

Nigeria is a multi-ethnic country and the cultural and religious inclination of different ethnic groups dictates the extent of interaction between adolescents of the opposite. Generally, the Nigerian society tends toward restrictive interactions between adolescents of the opposite sex but the norms are gradually changing and becoming more permissive. This varying cultural differences in the level of interactions between both sexes and its implication on PA was expressed by a participant: “*In fact, some culture would tell you that you are not supposed to interact with a male or a female* [which may be inevitable during sporting activities] *all those things are limiting them thereby impacting negatively on their health”* (FGD discussant from a Public Secondary School).

### Religious injunctions on female dressing with implication on sporting activity

Religious guidelines on female dressing which prohibits body revealing outfits was identified as a barrier to PA participation and a participant illustrated this with an example: *“Yes, I had an example during the last inter-house sport, this girl was so keen, she wanted to run. We had an external coach that came around and he told her that if she does not remove all these things* [clothes and garments which cover the body in line with Islamic injunctions], *he won’t allow her to run, well reluctantly she removed everything and she was begging “please I want to run” and by the time she did it, she came out well in-fact I was encouraged because I had thought that was the end*” (FGD discussant from a Public Secondary School).

### School-related factors influencing the physical activity behaviors of adolescents

This theme represents the school-related factors which influence the students’ physical activity behaviours within the school setting. Eight sub-themes were identified as barriers to physical activity among adolescents and the prominent ones were the declining number of trained PHE teachers, lack of PA equipment and facilities in schools, poor financing of schools, increased time devoted to the theoretical concepts rather than the practical PHE sessions, poor attitude of school stakeholders to PHE delivery and poor implementation of regulatory guide for the delivery of PHE. The eight sub-themes are discussed below:

#### Declining number of trained PHE teachers

This was a prominent sub-theme; according to the respondents, a lot of changes have occurred in the school settings over the years and these have detrimental effects on physical activity and exercise among adolescents. Of note is the decline in the number of trained Physical Health Education Specialist Teachers as expressed by a school Principal *“In times past, schools had at least one or two [PHE teachers] but now If you go to ten schools, I don’t think you can get more than two PHE teachers in all the ten schools”* Key Informant from a Public Secondary School. This finding according to participant had a detrimental effect for the delivery of PHE and the implementation of programmes to improve PA in schools.

#### Limited opportunities for continuing education for PHE teachers

This was another major prominent sub-theme as reported by the participants. There are limited opportunities for continuing education and professional development. This was due to the low prioritisation of PHE and invariably, those who teach this subject. Teachers for other subjects such as English and Mathematics more opportunities for continuing and professional development compared to PHE teachers as expressed by a school principal *“schools are very poor in staff development programmes because we believe seminars and workshop are not for PHE teachers, we just pick [choice] subjects. We hold seminars and workshops for Mathematic and English teachers, but for PHE teachers, we don’t” (*Key Informant from a Private Secondary School).

#### Lack of PA equipment and facilities

In most public and private schools, the delivery of physical health education classes was compromised due to the lack of equipment and sporting facilities, large student population without a corresponding number of PHE teachers as captured in this quote **“***Most schools lack the equipment* “(...)" *look at this school for example, since we’ve resumed this term, we have not had any PHE lesson, we don’t even have a PHE teacher”* (FGD Discussant in a Private School).

#### Limited availability of gender specific equipment and facilities for PA

Another pertinent factor identified as contributing to the low physical activity level of females is the limited gender specific physical activity facilities and equipment for sporting activities. Specific sports which female adolescents prefer include badminton, table tennis etc. but these are limited*: “why girls are not fully involved in PA?* “(...)" *it is because the facilities for the sport activities which girls like are costly. Girls enjoy sports such as badminton but the equipment are expensive* “(...)" *we can engage them to be active but we don’t have equipment for them [girls]. They also prefer table tennis which will not roughen their bodies [make them look rough] but these are expensive” (*FGD discussant from a Public School).

#### Increased time devoted to the theoretical concepts rather than the practical PHE sessions

Another major prominent sub-theme noted was the limited time for PHE classes. According to the respondents, the duration for each PHE classes was 40 min and there was more emphasis on the theoretical concepts than the practical sessions as expressed by a FGD participant from a public school *“the students take physical health education classes from Junior Secondary School 1 to 3. But they do more of the theoretical sessions than the practical and it is the fault of the government and the school administration”* Also noted was the fewer number of periods per week for PHE classes compared to other subjects *“the periods are not similar to what we have for subjects like Mathematics. If Mathematics comes up five times in a week, PHE may be once”* (Key Informant from a Private Secondary School).

#### Poor financing of schools

Financing was a major factor and sub-theme identified as limiting the availability of facilities and opportunities for PA in schools as illustrated by a Key informant in a public school *in fact let me tell you,* “(...)” *when you admit a student to the public school, they pay an annual tuition of N630 ($2), out of that amount, only 50 naira ($0.14) is allocated for sports. This is insufficient!*

#### Poor attitude of school stakeholders to PHE delivery

Compounding the problem is the poor attitude of teachers and school heads towards the delivery of physical health education and this was a major sub-theme. Some Teachers and school principals see PHE as a waste of time and are of the opinion this can be diverted to other subjects. Due to this, they are disinterested and don’t want to invest in the delivery of the subject: *“some School Principal and Teachers believe physical activity /physical education classes is a waste of time ... the time that the students spend jumping about and doing all those PA can be used for other meaningful subjects. At times, even we [Teachers] will say, what is Physical Health Education /PA? this is not necessary* “(...)“ *We also say what is ‘jumpology’? (a derogatory term for Physical Health Education) (scoffs) it is not necessary, we believe that Mathematics, English, Chemistry are important subjects but when it comes to PHE we believe it is not necessary and it is not important and that is why many schools don’t want to invest in it”* (FGD Discussant from a Private Secondary School).

#### Poor social support from teachers

The participant also reported poor social support from teachers for leisure physical activity in school. This they attributed to more interest in academic activities such as reading as well as the dislike of physical exercise: *“many teachers, they hinder and prevent the students from being active. Teachers usually prevent them and tell them to go and read even when it is time for them to do physical exercise. So many teachers think that reading, reading and reading only will help students”*. This view was supported by a key informant who strongly expressed her dislike for physical activity stating that *“if I see any opportunity where the student can be taken away from the field back in classroom, I would support such, you understand? I don’t like sports”* (Key Informant from a Private Secondary School).

#### Poor implementation of regulatory guide for the delivery of PHE

Majority of the respondents also expressed that despite some regulatory guides for the delivery of the PHE curriculum, the school management decide how and if they want PHE delivered in schools. It is easy for the schools to decide not to offer PHE since they know the parents place low value on the delivery of PHE compared to other subjects as expressed by a participant: “*the laws are there, PHE is compulsory, it is there but the parents, the school management [*school authorities] *and the society has already decided on what is paramount [i.e. other core subjects like mathematics and English] and what is not paramount [other subjects like physical health education]”(*FGD discussant from a Private Secondary School).

### Opportunities for physical activity promotion in schools

This theme summarises the extracurricular opportunities for the promotion of physical activity during school hours which include the assembly grounds, break time, after school and inter-house sporting competitions as well as the implementation challenges experienced with regards to these. All these sub-themes featured prominently in the data on the opportunities for physical activity promotion in schools.

#### Conduct of school assemblies

With regards to school assemblies, the respondents in public schools expressed that PA through this avenue is no longer possible. This was due to a policy directive from the Oyo State Ministry of Education to stop the conduct of school assemblies due to security threats as expressed in this quote: *“We’ve cancelled it [the School assembly]* “(...)" *we now conduct class assembly [where] they will pray, sing the national anthem, national pledge and the school anthem. Afterward, the normal class work will commence so we don’t have physical activity or sporting activity during that period” Key informant* from a Public Secondary School. However; private schools still utilise this opportunity as expressed by a key informant *“On Monday for instance we were on the assembly ground, we danced for an hour, acrobatic dancing, so we consider physical activity on the school assembly as being very important” Key Informant from a Private School.* This constitutes a missed opportunity for the promotion of PA in public schools.

#### Breaktime (recess)

Break time which is another feasible opportunity for PA has been severely constricted due to the introduction of new subjects which expanded the curricula and competing academic time. This presents a challenge to public but not private schools as stated in the quotes below “*I observe that the private schools have two break times - short break and long break, then it used to be the same in the public schools. However, we only have one period for the break* “(...)" *because we have so many subjects* “(...)" *some of these subjects are not useful*” FGD discussant from a Public Secondary School.

#### After school sessions

After school sessions can also help promote PA behaviours, however this opportunity is not explored due to the unwillingness of teachers to supervise activities during this period resulting in increased risk of accidents as expounded in this quote: *“immediately after school hours we expect them to leave the school compound and go home, we don’t want any casualties. Even during school hours, we have problems keeping an eye on them, several times they have fractured hand, broken legs and so on”* (FGD discussant from a Public Secondary School).

A participant shared an experience where participation in PA after school resulted in a causality*: “we had an experience sometimes ago. After school, the students arranged to go and swim. They went to the deepest part of the river and unfortunately, we lost the student. We always ensure that students leave the schools immediately just because of that experience*” (FGD discussant from a Public Secondary School).

#### Inter-house sports competition

Inter-house sports competition which holds potential for galvanising school-wide interest in PA was identified. According to the discussants in the private schools, inter-house sports competition hold yearly or every other year but the frequency of its conduct in public schools has reduced drastically due to lack of funding and the aftermath crisis associated with the failure of losing teams to accept defeat in the spirit of sportsmanship as expressed in this quote: *“most of the principals do not like conducting inter-house sports competition, during the inter-house sports competitions, students usually fight, that is the reason why most of the schools are not interested in the event”*(FGD discussant from a Public Secondary School).

Finally, of import is the negative impact of the poor road network, increased accident risks and government policies on the conduct of marathon and cross-country races as reflected in the quote below: *“previously before the inter-house sports or during the time of inter-house sports, we usually have cross country race but that is no more due to government policies and because of the poor connectivity of the road* “(...)“ *It’s not safe*” (FGD discussant from a Public Secondary School).

## Discussion

Findings from this qualitative study reveal that, the parental, socio-cultural and school-level factors influence the physical activity behaviors of adolescents thus suggesting the need for multi-level interventions to increase the opportunities for physical activity among adolescents. Other possible associated factors are religious and cultural standards which pressure females to conform to cultural norms by appearing feminine in order to be socially and culturally accepted thus limiting their participation in PA [37–39]. Oyeyemi et al. also observed this gender disparity in Northern Nigeria hence, there is a need to explore opportunities to positively influence norms associated with gender participation in sporting and physical activity in Nigeria. This also underscores the need for gender-specific interventions for physical activity promotion among Nigerian adolescents [10].

Social influences, attitude and support from significant others have implications on the physical activity behaviors of adolescents [13–15, 25, 40]. According to the respondents on this study, teachers especially those who teach other subjects aside physical health education generally have an unfavourable attitude towards the participation of adolescents in physical activity. This is viewed as a waste of time which can be devoted to other academic activities. Similar negative perceptions and attitudes have been reported in studies from other countries [13–15, 25] and have grave implication on the implementation and sustainability of physical activity policies and programmes in schools as the reality in schools provides little or no motivations to support or trigger adolescent’s ability to initiate and sustain PA. To address the physical activity barriers among adolescents, holistic interventions which address the negative attitudinal dispositions of teachers must be taken into consideration and prioritised.

The increasing demand for classroom academic time and pressures on education systems to improve students’ performance has had an unintended negative effect on the physical health education curricula thus reducing opportunities for physical activity in Nigeria [9, 20]. Findings from this study revealed that, most secondary schools offer physical education as an optional subject for pupils in the senior secondary level and few schools have PHE teachers to deliver the course thus, students can be exempted or excused. Similar findings have been reported in a study in Nigeria [9], and other countries [4, 41–43]. This underscores the need to develop a comprehensive national physical activity policy [24], and review and enforce the school curriculum on physical health education to ensure all students irrespective of their age and classes have opportunities to be active and attain their daily requirements.

Funding was a major factor identified as a barrier limiting the recruitment and availability of human resources, facilities and opportunities for PA in schools. According to the respondents, the number of trained Physical Health Education Specialist Teachers in public and private secondary schools have declined significantly and where they exist, their continuing education and professional development is not prioritised. In addition, findings from this study revealed the dearth of equipment and sporting facilities in the schools. These findings are similar to those obtained from studies in other countries [42, 44, 45]. This situation has compromised physical health education classes in schools in Nigeria resulting in an emphasize on the delivery of theoretical concepts to the in-school adolescents to the detriment of field based activities. Therefore, there is need for the government, school authorities and relevant stakeholders to increase financial investment to enhance human resources and improve the school environment equipment and facilities as a strategy to improve the physical activity behaviors of the adolescents.

There are limited extracurricular opportunities for the promotion of physical activity in public secondary schools during school hours. Physical activity during assembly on the school grounds has been cancelled due to a policy directive from the Oyo State Ministry of Education to stop its conduct due to security threats, instead assemblies are held in the classes. In addition, opportunities for inter-house sporting competitions have reduced due to sports-related violence especially brawling and security risk associated with competitive sporting events in public schools in Nigeria. Similar concern was reported in a study conducted among in-school adolescents in the north-central region of Nigeria [46]. This is a public health challenge that has been largely ignored [47]. To address this, there is need for multi-component interventions such community and school legislations to prevent violence during supporting events, environmental design of sporting venue and enforcement of sporting rules [47] to improve the school environment for physical activity. Thus, it is expedient for relevant sectors such as the Education, Youth and Sports and Security to work collaboratively to implement multisectoral interventions to provide safe and conducive environment for physical activity programmes and school-based sporting events among adolescents.

This study has contributed to the body of knowledge on barriers and opportunities for the promotion of PA for students within the school settings however, there were limitations. Firstly, though parents’ views were mentioned, the study did not include parents and adolescents in the group of participants interviewed. This would have provided a holistic, robust information on the barriers and facilitators for the promotion of PA among adolescents in line with the socio-ecological model. Secondly, though this study utilised the methodological triangulation approach i.e. the combined use of qualitative design to enhance understanding about the views of school principals and teachers about the physical activity behaviours of students however, we did not have multiple sources of information from each school which could have aided data triangulation, limited bias and enhanced data robustness. In view of these, studies which succinctly capture the multiple perspective of adolescents, parents and teachers on PA promotion within and outside the school settings are recommended for further studies.

## Conclusion

Findings from this study underscores the need for holistic, multi-component interventions which address the parental, socio-cultural and school-level factors which limits adolescents’ participation and opportunities for physical activity. There is a need to support the direct engagement of relevant school stakeholders to prioritise physical activity not only as a form of recreation but establish the current paradigm which stresses its linkages with overall physical and mental wellbeing. Of import is to strengthen government PA policy implementation at the school level through the involvement of policy actors in the overall intervention. There is a need to urgently promote and sustain the girl child participation in school-based physical activity, end the marginalisation and misconception of female participation in PA which is linked to cultural views about being muscular or losing virginity in order to mitigate the rising burden of NCDs among the future adults. Multi-sectoral interventions are needed to curtail the prevailing security threats in the country as well as brawling associated with competitive school-based sporting events. Finally, stakeholders at the institutional and policy levels need to prioritise the implementation of physical activity policies and programmes by increasing funding for the recruitment of teachers and provision of equipment and facilities in schools.

## Supplementary Information

**Additional file 1.** List of schools for the study.

**Additional file 2.** Key Informant interview guide for School Principals.

**Additional file 3.** Focus group discussion guide for teachers.

**Additional file 4.** Themes and sub-themes for qualitative data analysis.

## Data Availability

Transcripts can be provided upon request to the corresponding author. This can only be used for non-commercial purposes which ensures that participants’ confidentiality is protected.

## Abbreviations

FGD: Focus Group Discussion
GAPPA: Global Action Plan on Physical Activity
KII: Key Informant Interview
LGA: Local Government Area
MVPA: Moderate and Vigorous Physical Activity
NCDs: Non-Communicable Diseases
PA: Physical Activity
WHO: World Health Organisation

## References

[1] Ferreira de Moraes AC, Guerra PH, Menezes PR (2013). The worldwide prevalence of insufficient physical activity in adolescents; a systematic review. Nutr Hosp.

[2] Raitakari OT, Juonala M, Viikari JS (2005). Obesity in childhood and vascular changes in adulthood: insights into the cardiovascular risk in young Finns study. Int J Obes.

[3] Janssen I, LeBlanc AG (2010). Systematic review of the health benefits of physical activity and fitness in school-aged children and youth. Int J Behav Nutr Phys Act.

[4] Institute of Medicine. Educating the student body: Taking physical activity and physical education to school. Washington, DC: The National Academies Press; 2013. vol. 1. 24851299

[5] World Health Organisation. Global action plan on physical activity 2018–2030: more active people for a healthier world. Switzerland: World Health Organisation; 2018. Licence: CC BY-NC-SA 3.0 IGO.

[6] Guthold R, Stevens GA, Riley LM, Bull FC (2020). Global trends in insufficient physical activity among adolescents: a pooled analysis of 298 population-based surveys with 1·6 million participants. Lancet Child Adolesc Health.

[7] Odunaiya NA, Ayodele OA, Oguntibeju OO (2010). Physical activity levels of senior secondary school students in Ibadan, western Nigeria. West Indian Med J.

[8] Adeniyi AF, Okafor NC, Adeniyi CY (2011). Depression and physical activity in a sample of nigerian adolescents: levels, relationships and predictors. Child Adolesc Psychiatry Ment Health.

[9] Odunaiya NA, Grimmer K, Louw QA (2015). High prevalence and clustering of modifiable CVD risk factors among rural adolescents in Southwest Nigeria: implication for grass root prevention. BMC Public Health.

[10] Oyeyemi AL, Ishaku CM, Oyekola J, Wakawa HD, Lawan A, Yakubu S, et al. Patterns and associated factors of physical activity among adolescents in Nigeria. Plos One. 2016;11(2):1–16. 10.1371/journal.pone.0150142 PMC4762937 26901382

[11] Oyerinde OO, Oyesegun OO, Oshiname FO, Ola OO (2013). Knowledge of secondary school students in Ikenne lga, Ogun state, Nigeria on physical activity as a means of health promotion. Oman Chapter Arab J Bus Manag Rev.

[12] Senbanjo IO, Oshikoya KA (2010). Physical activity and body mass index of school children and adolescents in Abeokuta. Southwest Niger World J Pediatr.

[13] Musaiger AO, Al-Mannai M, Tayyem R, Al-Lalla O, Ali EY, Kalam F, et al. Perceived barriers to healthy eating and physical activity among adolescents in seven Arab countries: a cross-cultural study. Sci World J. 2013;Article ID 232164:1-16. 10.1155/2013/232164 PMC3848306 24348144

[14] Martins J, Marques A, Sarmento H (2015). Carreiro da Costa F. adolescents’ perspectives on the barriers and facilitators of physical activity: a systematic review of qualitative studies. Health Educ Res.

[15] Nathan N, Elton B, Babic M, McCarthy N, Sutherland R, Presseau J, Seward K, Hodder R, Booth D, Yoong SL, Wolfenden L (2018). Barriers and facilitators to the implementation of physical activity policies in schools: a systematic review. Prev Med.

[16] Huberty J, Dinkel D, Coleman J, Beighle A, Apenteng B (2012). The role of schools in children’s physical activity participation: staff perceptions. Health Educ Res.

[17] Sallis JF, Cervero RB, Ascher W, Henderson KA, Kraft MK, Kerr J (2006). An ecological approach to creating active living communities. Annu Rev Public Health.

[18] Akinroye KK, Adeniyi AF (2018). Results from Nigeria’s 2018 report card on physical activity for children and youth. J Phys Act Health.

[19] Ojo AL (2015). Teaching physical education in Nigerian secondary schools is a barrier: an implication for future generation, a case study of ado metropolis secondary schools in Ekiti state. Niger Int J Educ Learn Dev.

[20] Edim ME, Ogabor JO, Odok EA (2014). Adaptability of physical education curriculum in schools in Nigeria: the way forward. Int J Capacit Build Educ Manag.

[21] National Sports Commission (2009). National Sports Policy of Nigeria.

[22] Federal Ministry of Education (2006). N a t i o n a l S c h o o l H e a l t h P o l i c y.

[23] Federal Ministry of Health (2019). National Multi-Sectoral Action Plan for the Prevention and Control of Non-communicable Diseases (2019-2025).

[24] Oluwasanu M, Oladunni O, Oladepo O. Multisectoral approach and WHO ‘Bestbuys’ in Nigeria’s nutrition and physical activity policies. Health Promot Int. 2020;35:1383–93. 10.1093/heapro/daaa009 32087010

[25] Morton KL, Atkin AJ, Corder K, Suhrcke M, van Sluijs EM (2016). The school environment and adolescent physical activity and sedentary behaviour: a mixed-studies systematic review. Obes Rev.

[26] Hynynen ST, Van Stralen MM, Sniehotta FF, Araújo-Soares V, Hardeman W, Chinapaw MJ, Vasankari T, Hankonen N (2016). A systematic review of school-based interventions targeting physical activity and sedentary behaviour among older adolescents. Int Rev Sport Exerc Psychol.

[27] Wechsler H, Devereaux RS, Davis M, Collins J (2000). Using the school environment to promote physical activity and healthy eating. Prev Med.

[28] Verstraete SJ, Cardon GM, De Clercq DL, De Bourdeaudhuij IM (2006). Increasing children's physical activity levels during recess periods in elementary schools: the effects of providing game equipment. Eur J Pub Health.

[29] Jago R, Baranowski T (2004). Non-curricular approaches for increasing physical activity in youth: a review. Prev Med.

[30] US Department of Health and Human Services. Physical Activity Guidelines Advisory Committee. In: Physical activity guidelines advisory committee report. Washington, DC; 2008. p. A1–H14. https://www.europarc.org/wp-content/uploads/2018/03/Physical-Activity-Guidelines-Advisory-Committee-Report-2008.pdf.

[31] McLeroy KR, Bibeau D, Steckler A, Glanz K (1988). An ecological perspective on health promotion programmes. Health Educ Q.

[32] Oluwasanu MM, Oladepo O (2017). Effects of a multi-level intervention on the pattern of physical activity among in-school adolescents in Oyo state Nigeria: a cluster randomised trial. BMC Public Health.

[33] Denzin NK. The research act: a theoretical introduction to sociological methods: Transaction publishers; 2017.

[34] Fusch P, Fusch GE, Ness LR (2018). Denzin’s paradigm shift: revisiting triangulation in qualitative research. J Soc Change.

[35] Smith LF, Eve R, Crabtree R (2000). Higher professional education for general medical practitioners: key informant interviews and focus group findings. Br J Gen Pract.

[36] Lo BK, Morgan EH, Folta SC, Graham ML, Paul LC, Nelson ME, Jew NV, Moffat LF, Seguin RA (2017). Environmental influences on physical activity among rural adults in Montana, United States: views from built environment audits, resident focus groups, and key informant interviews. Int J Environ Res Public Health.

[37] Craig E, Bland R, Reilly J (2013). Objectively measured physical activity levels of children and adolescents in rural South Africa: high volume of physical activity at low intensity. Appl Physiol Nutr Metab.

[38] Cockburn C, Clarke G (2002). “Everybody’s looking at you!”: girls negotiating the “femininity deficit” they incur in physical education. Women’s studies international forum.

[39] Witmer L, Bocarro JN, Henderson K (2011). Adolescent girls’ perception of health within a leisure context. J Leis Res.

[40] Martins J, Marques A, Peralta M, Palmeira A, Da Costa FC (2017). Correlates of physical activity in young people. Retos-Nuevas Tendencias En Educacion Fisica Deporte Y Recreacion.

[41] Boyle SE, Jones GL, Walters SJ (2008). Physical activity among adolescents and barriers to delivering physical education in Cornwall and Lancashire, UK: a qualitative study of heads of PE and heads of schools. BMC Public Health.

[42] Hassan AR, Rashid MH, Smith G, Selim MA, Rasheed S (2020). Challenges of promoting physical activity among school children in urban Bangladesh: a qualitative inquiry. PLoS One.

[43] Hills AP, Dengel DR, Lubans DR (2015). Supporting public health priorities: recommendations for physical education and physical activity promotion in schools. Prog Cardiovasc Dis.

[44] Tarun S, Arora M, Rawal T, Benjamin Neelon SE (2017). An evaluation of outdoor school environments to promote physical activity in Delhi, India. BMC Public Health.

[45] Parobii I, Springer AE, Harrell MB, Gomensoro LM, Fresco MT, Alers N, Perry CL, Estol D (2018). Exploring physical activity engagement in secondary school students in Montevideo, Uruguay: a qualitative study. Int J Child Adolesc Health.

[46] Owojaiye SO, Bitrus M (2015). Development of sports administrators for enhancement of physio psycho-social health security of Nigerian youths, journal of education. Arts Humanities.

[47] Fields SK, Collins CL, Comstock RD (2010). Violence in youth sports: hazing, brawling and foul play. Br J Sports Med.

