# Supplementary material for: Qualitative views of Nigerian school principals and teachers on the barriers and opportunities for promoting students’ physical activity behaviours within the school settings
Source: BMC Public Health. 2021 Dec 19;21:2302. doi: 10.1186/s12889-021-12327-x (PMC8684632; doi:10.1186/s12889-021-12327-x)
Supplement: Supplementary file 1 — Additional file 1. List of schools for the study. [file 12889_2021_12327_MOESM1_ESM.doc]

| **S.No** | **Name of Schools** | |  | **Contribution to data collection** | **Type** | **Student type** | **Physical** | |  | **Availability** | | **of** |
| --- | --- | --- | --- | --- | --- | --- | --- | --- | --- | --- | --- | --- |
|  |  |  |  |  |  |  | **Health** | |  | **sporting** | |  |
|  |  |  |  |  |  |  | **Education** | |  | **facilities** | |  |
|  |  |  |  |  |  |  | **in School** | |  |  |  |  |
|  |  |  |  |  |  |  |  |  |  |  |  |  |
| 1. | School 1 |  |  | Key informant interview with school Principal | Public | Co-educational | Yes, | but | not | Open | field | and |
|  |  |  |  |  |  |  | taught | at the | | basic | equipment | |
|  |  |  |  |  |  |  | time | of | the |  |  |  |
|  |  |  |  |  |  |  | study due to the | | |  |  |  |
|  |  |  |  |  |  |  | lack of PHE | | |  |  |  |
|  |  |  |  |  |  |  | teachers | |  | i.e. footballs | |  |
|  |  |  |  |  |  |  |  |  |  |  | study |  |
| 2. | School 2 | | | Focus group discussion (10 teachers) | Public | Co-educational | Yes |  |  | Open | field | and |
|  |  | |  |  |  |  |  |  |  | basic equipment | | |
|  |  |  |  |  |  |  |  |  |  | i.e. footballs and | | |
|  |  |  |  |  |  |  |  |  |  | table tennis | |  |
|  |  |  |  |  |  |  |  |  |  |  |  |  |
| 3. | School 3 |  |  | Focus group discussion (9 teachers) | Public | Co-educational | Yes |  |  | Open | field | and |
|  |  |  |  |  |  |  |  |  |  | basic | equipment | |
|  |  |  |  |  |  |  |  |  |  | i.e. football | |  |
|  |  |  |  |  |  |  |  |  |  |  |  |  |
| 4. | School 4 |  |  | Key informant interview | Public | Co-educational | Yes |  |  | Open | field | and |
|  |  | |  |  |  |  |  |  |  | basic | equipment | |
|  |  |  |  |  |  |  |  |  |  | i.e. footballs | |  |
|  |  |  | |  |  |  |  |  |  |  |  |  |
| 5. | School 5 |  | | Key informant interview with school Principal | Private | Co-educational | Yes |  |  | Open | field | and |
|  |  | |  |  |  |  |  |  |  | basic | equipment | |
|  |  |  |  |  |  |  |  |  |  | i.e. | footballs, | |
|  |  |  |  |  |  |  |  |  |  | table tennis | |  |
|  |  |  | |  |  |  |  |  |  |  |  | |
| 6. | School 6 |  | | Focus group discussion (8 teachers) | Private | Co-educational | Yes |  |  | Well | equipped | |
|  |  |  |  |  |  |  |  |  |  | with a field/pitch | | |
|  |  |  |  |  |  |  |  |  |  | for football | | and |
|  |  |  |  |  |  |  |  |  |  | basketball | |  |
|  |  | | |  |  |  |  |  |  |  |  |  |

| 7. | School 7 | | | Key informant interview with school Principal | Public | Co-educational | Yes |  |  | Open | field | and |
| --- | --- | --- | --- | --- | --- | --- | --- | --- | --- | --- | --- | --- |
|  |  |  |  |  |  |  |  |  |  | basic | equipment | |
|  |  |  |  |  |  |  |  |  |  | i.e. football | |  |
|  |  | |  |  |  |  |  |  |  |  |  |  |

| 8. | School 8 | |  | Focus group discussion (9 teachers) | Public | Co-educational | Yes |  |  | Open | field | and |
| --- | --- | --- | --- | --- | --- | --- | --- | --- | --- | --- | --- | --- |
|  |  |  |  |  |  |  |  |  |  | basic | equipment | |
|  |  |  |  |  |  |  |  |  |  | i.e. football | |  |
|  |  |  |  |  |  |  |  | | |  |  |  |
|  |  |  |  |  |  |  | Yes but not | | |  |  |  |
|  |  |  |  |  |  |  | taught at the time | | |  |  |  |
|  |  |  |  |  |  |  | of the study due | | |  |  |  |
|  |  |  |  |  |  |  | to the lack of | | |  |  |  |
| 9. | School 9 |  | | Focus group discussion (9 teachers) | Public | Co-educational | PHE teachers | | | Open | field | and |
|  |  |  |  |  |  |  |  |  |  | basic | equipment | |
|  |  |  |  |  |  |  |  |  |  | i.e. football | |  |
|  |  |  |  |  |  |  |  |  |  |  |  |  |

| 10. | School 10 |  | Key informant interview with school Vice Principal | Public | Co-educational | Yes | Open | field | and |
| --- | --- | --- | --- | --- | --- | --- | --- | --- | --- |
|  |  |  |  |  |  |  | basic | equipment | |
|  |  |  |  |  |  |  | i.e. football | |  |
|  |  | |  |  |  |  |  | |  |
| 11. | School 11 | | Key informant interview with school Principal | Private | Co-educational | Yes | Well equipped | |  |
|  |  |  |  |  |  |  | with a field/pitch | | |
|  |  | |  |  |  |  | for football | |  |
|  |  |  |  |  |  |  |  |  |  |
| 12. | School 12 |  | Focus group discussion (8 Teachers) | Private | Co-educational | No | Open | field | and |
|  |  |  |  |  |  |  | basic | equipment | |
|  |  |  |  |  |  |  | i.e. football | |  |
|  |  |  |  |  |  |  |  |  |  |
