# Supplementary material for: Qualitative views of Nigerian school principals and teachers on the barriers and opportunities for promoting students’ physical activity behaviours within the school settings
Source: BMC Public Health. 2021 Dec 19;21:2302. doi: 10.1186/s12889-021-12327-x (PMC8684632; doi:10.1186/s12889-021-12327-x)
Supplement: Supplementary file 2 — Additional file 2. Key Informant interview guide for School Principals. [file 12889_2021_12327_MOESM2_ESM.pdf]

IN-DEPTH INTERVIEW GUIDE FOR SCHOOL PRINCIPALS

	Question	Probes
1.	What do you think about the health of in-school adolescents in your school?	*Probe for*<br><br>*Common diseases*<br>*Health behaviours specifically physical activity,*
2.	In your view, what is the level of physical activity among secondary school students/pupils in your school	Probe if<br><br>High or Low based on age (i.e. 10-14 and 15-19) and gender (i.e. males or females)<br>In-school adolescents attain the recommended daily target of 60 minutes of Moderate to Vigorous Physical activity
3.	What do you think about the opportunities that students have to engage in physical activity during school hours	Probe for<br><br>Frequency and opportunities for engagement in structured physical activity (i.*e. sports, class based activities, regular school physical activity programmes*)<br>Frequency and opportunities for unstructured activities (*i.e. break time, after school programmes etc*)<br>Use of physical activity as a form of corporal punishment
4.	In your view, are there school guidelines that determine the extent to which students have opportunities for physical activity	Probe for the availability, use and compliance to<br><br>*School Time table*<br>*Curriculum for Physical Health Education*
5.	What factors influence the physical activity behaviours of in-school adolescents	Probe for<br><br>Social norms including the attitude of students and teachers,<br>Social support,<br>Cultural factors especially gender issues<br>Religious factors<br>Physical/built environment,<br>Availability of equipment and facilities<br>School guidelines such as *School Time table, Curriculum for Physical Health Education, School inspectors who monitor activities of school/scheme of work in line with the curriculum*

6.	What are the barriers to the implementation of Physical activity in schools	Probe for<br><br>Attitude of students<br>Competing academic time<br>Social norms and support(i.e. attitude of teachers, *parents, Parent Teachers Association etc*)<br>Availability of Physical and Health Education staff<br>Staff development programmes for PHE teachers<br>*Availability of equipment and facilities*<br>*Guidelines on physical activity in school specifically, School Time table, Curriculum for Physical Health Education*<br>Funding and budget allocation for physical education equipment, programmes and supplies?
7.	What are the recommended activities which have the potentials of ensuring in-school adolescents attain the daily target of 60 minutes of moderate and vigorous daily activity	Probe for the appropriate interventions/activities to improve physical activity in schools<br><br>Probe for the most appropriate time and setting
8.	What other actions would you recommend to facilitate physical activity in schools	*Provision of educational materials (i.e. posters and handbills) for students, Film show on different types of physical activity, Special Dance events in schools, Experience sharing by role models (i.e. Athletes), M health – sending text messages (SMS reminders), Training programme for students on how to be physically active ,Empowering students to develop fun activities aimed at promoting PA and others"*
