# Supplementary material for: Qualitative views of Nigerian school principals and teachers on the barriers and opportunities for promoting students’ physical activity behaviours within the school settings
Source: BMC Public Health. 2021 Dec 19;21:2302. doi: 10.1186/s12889-021-12327-x (PMC8684632; doi:10.1186/s12889-021-12327-x)
Supplement: Supplementary file 3 — Additional file 3. Focus group discussion guide for teachers. [file 12889_2021_12327_MOESM3_ESM.pdf]

FOCUS GROUP DISCUSSION GUIDE FOR SUBJECT/CLASS TEACHERS

	Question	Probes
1.	Health of in-school adolescents	*Probe for*<br><br>*Common diseases*<br>*Health behaviours specifically physical activity*
2.	Level of physical activity among students	Probe if<br><br>High or Low based on age (i.e. 10-14 and 15-19) and gender (i.e. males or females)<br>In-school adolescents attain the recommended daily target of 60 minutes of Moderate to Vigorous Physical activity
3.	Opportunities that students have to engage in physical activity during school	Probe for<br><br>Frequency and opportunities for engagement in structured physical activity (i.*e. sports, class based activities, regular school physical activity programmes*)<br>Frequency and opportunities for unstructured activities (*i.e. break time, after school programmes etc*)<br>Type of PAs students engage in' Probe for those liked /those not liked<br>What students consider as appropriate/inappropriate PAs"<br><br>Use of physical activity as a form of corporal punishment
4.	Factors promoting or hindering physical activity behaviours of adolescents in school settings	Probe for<br><br>Secondary school students thoughts about physical exercise in schools'<br>Social norms including the attitude parents, teachers and school authorities<br>Cultural factors especially gender issues<br>Religious factors<br>Physical/built environment,<br>Availability of equipment and facilities<br>Policies on physical activity in school *specifically the School Health policy, School Time table reflecting the break time, Curriculum for Physical Health Education*

		Role of government, teachers, parents, community, others’
5.	Recommended activities which have the potentials of ensuring in-school adolescents attain the daily target of 60 minutes of moderate and vigorous daily activity	Probe for the appropriate interventions/activities to improve physical activity in schools<br><br>Probe for the most appropriate time and setting
6.	Recommended actions to facilitate physical activity in the schools	
