# Supplementary material for: Qualitative views of Nigerian school principals and teachers on the barriers and opportunities for promoting students’ physical activity behaviours within the school settings
Source: BMC Public Health. 2021 Dec 19;21:2302. doi: 10.1186/s12889-021-12327-x (PMC8684632; doi:10.1186/s12889-021-12327-x)
Supplement: Supplementary file 4 — Additional file 4. Themes and sub-themes for qualitative data analysis. [file 12889_2021_12327_MOESM4_ESM.docx]

**Themes and Sub themes on the Qualitative views of Nigerian School Principals and Teachers on their Students’ Physical Activity Behaviors**

**Theme: Perceived parental factors influencing the physical activity behaviors of adolescents**

**Sub themes:**

- Parental education and participation in PA
- Preference for motorized transportation
- Cultural beliefs on PA and fertility

## Theme: Socio-cultural and religious factors

**Sub themes:**

- Cultural beliefs on effect of PA on female anatomy and health
- Religious injunctions on female dressing
- Cultural limitations on opposite sex interactions

## Theme: School-related factors

**Sub themes**:

- Declining number of trained PHE teachers
- Limited opportunities for continuing education
- Limited availability of PA equipment and facilities
  - Limited availability of gender specific equipment and facilities
- Increased time devoted for PHE theoretical concepts
- Poor financing of schools
- Poor attitude of school stakeholders to PHE delivery
- Poor social support from Teachers
- Poor implementation of PHE curriculum

## Theme: Opportunities for physical activity promotion in schools

**Sub themes:**

**School assemblies**

- School assembly and security threat

**Breaktime (recess)**

- Competing academic time

**After school sessions**

- Poor supervision and causalities

**Inter house sports competition**

- Poor funding
- Hooliganism
- Poor road network
